# A chromosome-scale genome assembly of a diploid alfalfa, the progenitor of autotetraploid alfalfa

**DOI:** 10.1038/s41438-020-00417-7

**Published:** 2020-12-01

**Authors:** Ao Li, Ai Liu, Xin Du, Jin-Yuan Chen, Mou Yin, Hong-Yin Hu, Nawal Shrestha, Sheng-Dan Wu, Hai-Qing Wang, Quan-Wen Dou, Zhi-Peng Liu, Jian-Quan Liu, Yong-Zhi Yang, Guang-Peng Ren

**Affiliations:** 1 State Key Laboratory of Grassland Agro-Ecosystems, Institute of Innovation Ecology & School of Life Sciences, Lanzhou University, Lanzhou, China; 2 Key Laboratory of Adaptation and Evolution of Plateau Biota, Northwest Institute of Plateau Biology, Chinese Academy of Sciences, Xining, China; 3 State Key Laboratory of Grassland Agro-Ecosystems, Key Laboratory of Grassland Livestock Industry Innovation, Ministry of Agriculture and Rural Affairs, College of Pastoral Agriculture Science and Technology, Lanzhou University, Lanzhou, China; 4 Key Laboratory of Bio-Resources and Eco-Environment of the Ministry of Education & State Key Lab of Hydraulics & Mountain River Engineering, College of Life Sciences, Sichuan University, Chengdu, China

**Keywords:** Genome, Evolution

## Abstract

Alfalfa (*Medicago sativa* L.) is one of the most important and widely cultivated forage crops. It is commonly used as a vegetable and medicinal herb because of its excellent nutritional quality and significant economic value. Based on Illumina, Nanopore and Hi-C data, we assembled a chromosome-scale assembly of *Medicago sativa* spp*. caerulea* (voucher PI464715), the direct diploid progenitor of autotetraploid alfalfa. The assembled genome comprises 793.2 Mb of genomic sequence and 47,202 annotated protein-coding genes. The contig N50 length is 3.86 Mb. This genome is almost twofold larger and contains more annotated protein-coding genes than that of its close relative, *Medicago truncatula* (420 Mb and 44,623 genes). The more expanded gene families compared with those in *M. truncatula* and the expansion of repetitive elements rather than whole-genome duplication (i.e., the two species share the ancestral Papilionoideae whole-genome duplication event) may have contributed to the large genome size of *M. sativa* spp*. caerulea*. Comparative and evolutionary analyses revealed that *M. sativa* spp*. caerulea* diverged from *M. truncatula* ~5.2 million years ago, and the chromosomal fissions and fusions detected between the two genomes occurred during the divergence of the two species. In addition, we identified 489 resistance (*R*) genes and 82 and 85 candidate genes involved in the lignin and cellulose biosynthesis pathways, respectively. The near-complete and accurate diploid alfalfa reference genome obtained herein serves as an important complement to the recently assembled autotetraploid alfalfa genome and will provide valuable genomic resources for investigating the genomic architecture of autotetraploid alfalfa as well as for improving breeding strategies in alfalfa.

## Introduction

**Fig. 1:**
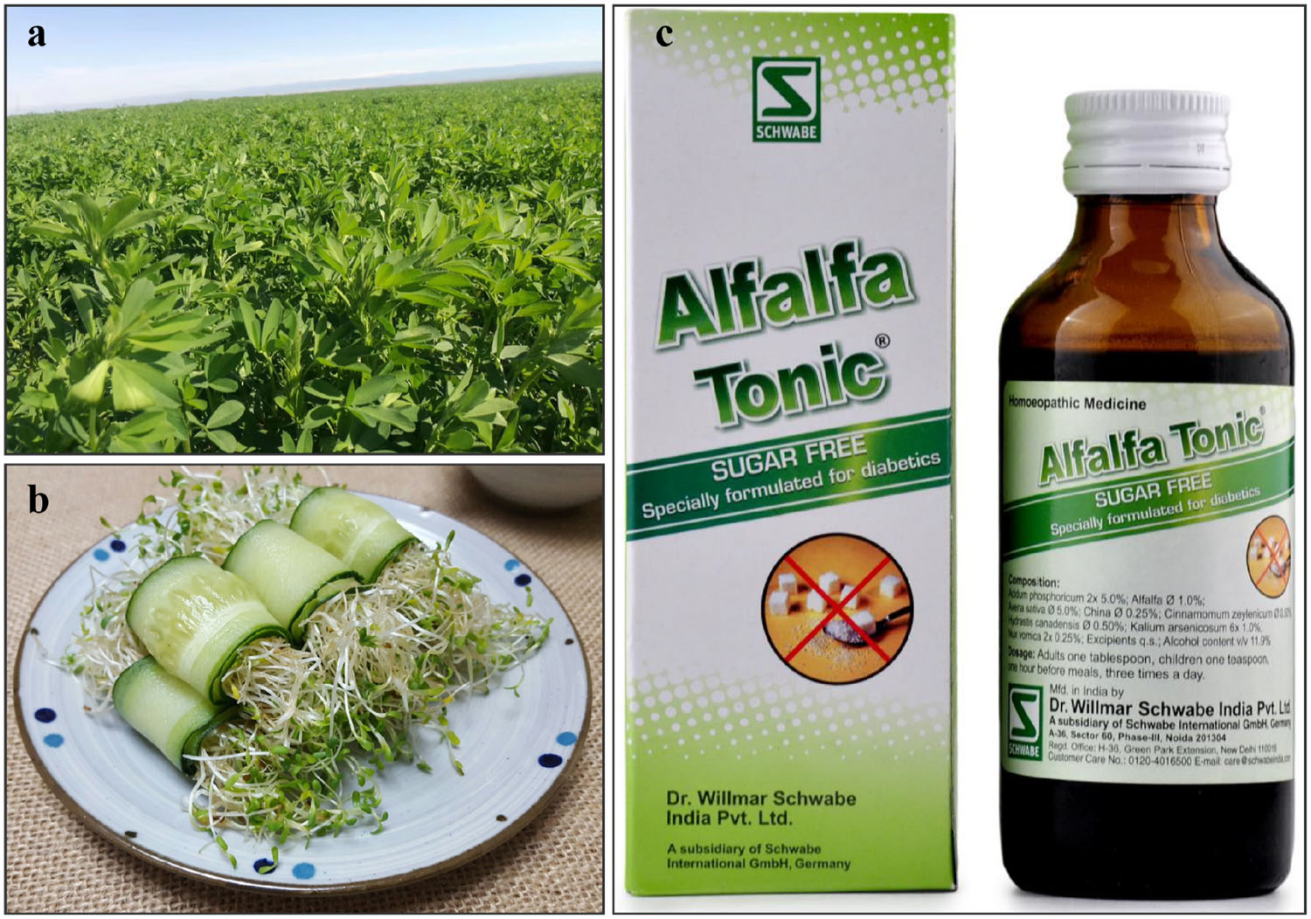
Photographs of alfalfa uses and products. **a** Photograph of alfalfa used as a widely cultivated forage crop. **b** Photograph of alfalfa as food. **c** Photograph of alfalfa as a health product. Picture from Dr. Willmar Schwabe, India Pvt., Ltd.

Alfalfa (*Medicago sativa* ssp*. sativa* L.) is a perennial legume forage that is widely cultivated for hay, pasture and silage production (e.g., Fig. 1a). As one of the most economically valuable crops in the world^1–3^, alfalfa has total estimated annual sales ranging from 7.8 to 10.8 billion dollars in the USA^4^. Alfalfa is known as “the queen of forage crops” not only because of its high-protein content and nutritive value as an animal feed but also because of its atmospheric nitrogen (N) fixation capacity. It is used as a rotation crop to increase soil fertility and serves as an important habitat for wildlife^5^. In addition, alfalfa is well known for its superior contents of vitamins (A, C, E, and K), protein and minerals, such as calcium, potassium, phosphorus, and iron^6^. Its seed sprouts and tender tips contain all these nutrients but few calories and are often used as edible vegetables (e.g., Fig. 1b). Furthermore, alfalfa has long been used as a medicinal herb. Its seeds or dried leaves can be used as a nutritional supplement and are sold as a bulk powdered herb, capsules, and tablets in health food stores^7^. The extracts from alfalfa seeds and leaves have hypocholesterolemic, neuroprotective, antioxidant, hypolipidemic, and antimicrobial effects and are used in the treatment of diabetes, stroke, cancer and menopausal symptoms^6,8–12^ (e.g., Fig. 1c). Alfalfa also exhibits a relatively high level of disease resistance potential compared to that of other food crops^13^. Therefore, it provides disease prevention between planting stages and increases the stock carrying capacity. In China, the cultivated area of alfalfa reached 3.6 million hectares in 2017; however, China still imports more than 1.3 million tons per year, accounting for ~85% of the total imported hay. An increasing industrial demand, low production and a lack of multiple improved varieties with strong resistance and quality may be some of the factors accounting for such a large supply gap in the alfalfa industry^14^.

On the basis of advanced sequencing technologies, breeders can use DNA markers combined with genome sequences to facilitate gene discovery, trait dissection and predictive molecular breeding technology^15,16^. Despite the high economic value of and increasing industrial demand for alfalfa, improvements through breeding are very limited, partly due to a lack of information on the whole genome. Alfalfa is suggested to be an autotetraploid (2*n* = 4*x* = 32) subspecies in the *M. sativa* complex^17,18^. The recently published genome assembly of an autotetraploid alfalfa^19^ is expected to greatly facilitate the future improvement of molecular breeding strategies. However, assembling a complete autotetraploid genome is still challenging due to essential features of tetrasomic inheritance, as more than 400 Mb of contigs were not placed onto the chromosomes in the above genome assembly^19^. In this case, assembling the genome of the diploid progenitor could be an alternative way to obtain full genomic information for alfalfa. Indeed, genomic information for diploid progenitors has provided substantial insights into selection for several key agronomic traits and the evolutionary history of multiple polyploid food crops, such as cotton^20^, wheat^21^, and strawberry^22^.

Previous studies have demonstrated that *M. sativa* spp*. caerulea* (2*n* = 2*x* = 16), a perennial self-incompatible herb, is the diploid progenitor of autotetraploid alfalfa^23^. In this study, we assembled a chromosome-scale draft genome of *M. sativa* spp*. caerulea* voucher PI464715 (hereafter PI464715) using a combination of Illumina, Hi-C and Nanopore sequencing technologies. Using this high-quality genome, we further performed genome annotation, evolutionary analysis, and comparative genomics and identified resistance genes and genes involved in the lignin and cellulose biosynthesis pathways. Our PI464715 genome assembly provides a diploid reference for analyzing the alfalfa genome and is a valuable resource for future molecular breeding of alfalfa. This genome is also beneficial for investigating genome evolution in the genus *Medicago* and related taxa.

## Results

### Genome sequence and assembly

*Medicago sativa* spp*. caerulea* (voucher PI464715; 2*n* = 2*x* = 16) was chosen for genome sequencing and assembly. A genome survey was first performed to assess the genome size based on 81.5 Gb of Illumina data. Using K-mer analysis, we evaluated the genome size to be ~802 Mb, with a high level of heterozygosity of 1.9% (Supplementary Table S1 and Supplementary Fig. 1). To accurately assemble this highly heterozygous genome, Illumina, Nanopore and Hi-C technologies were adopted for sequencing, and a series of methods were performed for assembly. Based on 116.5 Gb of Nanopore long reads corresponding to ~145× coverage of the estimated ~802 Mb genome, we preliminarily obtained a raw assembled genome of 1,345.8 Mb and contig N50 of 2.8 Mb by the NextGraph module. After polishing by NextPolish^24^ and performing deredundancy by purge_haplotigs^25^, we obtained the final genome assembly with a length of 793.2 Mb and a contig number of 355 and contig N50 of 3.86 Mb, constituting 98.9% of the predicted genome size (Table 1; Supplementary Table S1). We used the Benchmarking Universal Single-Copy Orthologs (BUSCO) evaluation score^26^ to assess the quality of the assembly, which resulted in 97.7% gene set completeness (Supplementary Table S2), indicating a very complete and high-quality genome assembly. We further connected 338 (95.2%) out of 355 contigs into eight pseudochromosomes based on ~224 Gb of Hi-C data (~279×coverage) using the hierarchical clustering strategy^27^ (Supplementary Fig. 2; Supplementary Tables S1 and S3). In total, 98.5% (781 Mb) of the assembly was anchored and oriented on eight pseudochromosomes, which ranged from 83.24 to 118.42 Mb in length (Supplementary Table S3), and 98.3% of transcriptomic reads and 96.4% of Illumina short reads could be properly mapped to the final genome assembly (Supplementary Tables S4 and S5).

**Table 1:** Summary of the PI464715 genome assembly and annotation.

Categories	Type	Length (bp)	No.	% of genome
Assembly	Contigs	793,191,298	355	-
	Contig N50	3,857,628	65	-
	Contig N90	1,207,163	205	-
	Longest	14,548,009	1	-
Noncoding RNAs	miRNA	117,689	1023	0.015
	snRNA	269,590	2438	0.034
	rRNA	331,884	1978	0.042
	tRNA	63,912	857	0.008
Transposable elements	DNA	57,410,696	-	7.24
	LINE	34,454,045	-	4.34
	SINE	2,527,147	-	0.32
	LTR	251,835,738	-	31.75
	RC	15,102,186	-	1.9
	Satellite	342,023	-	0.043
	Simple_Repeat	10,038,485	-	1.26
	Unknown	80,412,452	-	10.14
	Low_Complexity	1,803,306	-	0.23
	Total	440,637,371	-	55.55
Gene	Gene loci	-	47,202	-
	Average gene length (bp)	3151	-	-
	Average CDS length (bp)	1085	-	-
	Average exon length (bp)	231	-	-
	Average exons per gene	-	4.7	-
	Average intron length	615	-	-

Our PI464715 assembly provided significant improvement (with larger contig sizes and a higher BUSCO score) than the alfalfa genome^19^. Our genome has a contig N50 of 3.86 Mb, which is ~8.4-fold greater than that of the alfalfa genome (459 kb). Moreover, we placed 781 Mb of the assembly onto eight chromosomes with the aid of Hi-C data, while in the alfalfa genome, only 685 Mb (on average across the four assembled monoploid genomes) was anchored on the eight chromosomes. Our assembled genome also obtained a higher BUSCO evaluation score (97.7%) than the four monoploid genomes of alfalfa (88.5%, 88.3%, 87.5%, and 87.2%). All these comparisons indicated that our genome has better contiguity and higher quality.

### Gene prediction and annotation

**Fig. 2:**
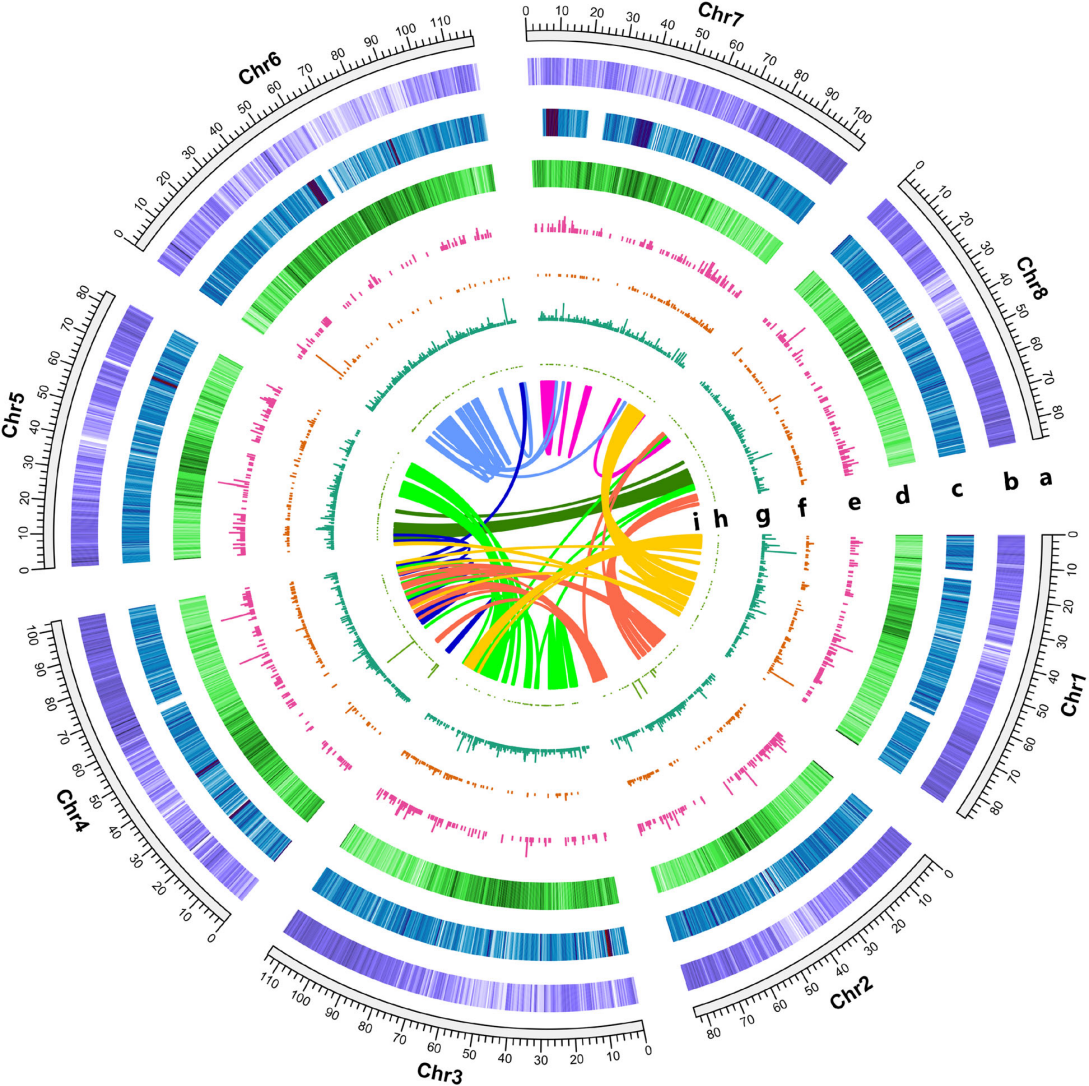
Overview of the PI464715 genome assembly. **a** Genomic positions (in Mb) of the eight chromosomes of PI464715. **b** Gene density. **c** Transposable element (TE) content. **d** Guanine-cytosine (GC) content. **e** miRNA density. **f** tRNA density. **g** snRNA density. **h** rRNA density. **i** Interconnections of paralogs in the genome are represented with colored lines.

In total, we identified 47,202 protein-coding genes, with an average gene length of 3151 bp (Table 1 and Supplementary Fig. 3), based on a combined strategy using de novo, transcriptome-based and homology-based methods. The total GC content of the PI464715 genome assembly was 34.21% (Supplementary Table S3). BUSCO evaluation further showed that the annotated PI464715 genome contained 97% BUSCOs (Supplementary Table S6). Then, five protein databases, namely, InterPro, Kyoto Encyclopedia of Genes and Genomes (KEGG), SwissProt, KOG and NR, were used to compare our protein models. Overall, we assigned potential functions to 92.51% (43,669) of the protein-coding genes in the PI464715 genome (Supplementary Table S7). The gene distribution and GC content along each chromosome were calculated, and their distributions were uneven (Fig. 2b, d), as also found in many other plant species (e.g.*, M. truncatula*). In addition, we identified 857 transfer RNAs (tRNAs), 1023 microRNAs (miRNAs), 1978 ribosomal RNAs (rRNAs), and 2438 small nuclear RNAs (snRNAs) in the PI464715 genome (Table 1 and Fig. 2e–h).

We annotated repetitive sequences of the genome using both de novo and homology-based approaches. We annotated ~440 Mb (55.55%) of the PI464715 genome assembly that comprised transposable elements (TEs), of which long terminal repeat (LTR) retrotransposons were the most abundant, accounting for 57.2% of TEs and 31.75% of the assembled genome (Fig. 2c and Table 1). DNA transposons, long interspersed nuclear elements (LINEs) and short interspersed nuclear elements (SINEs) accounted for 7.24%, 4.34%, and 0.32% of the genome assembly, respectively (Table 1).

### Gene-family analysis

**Fig. 3:**
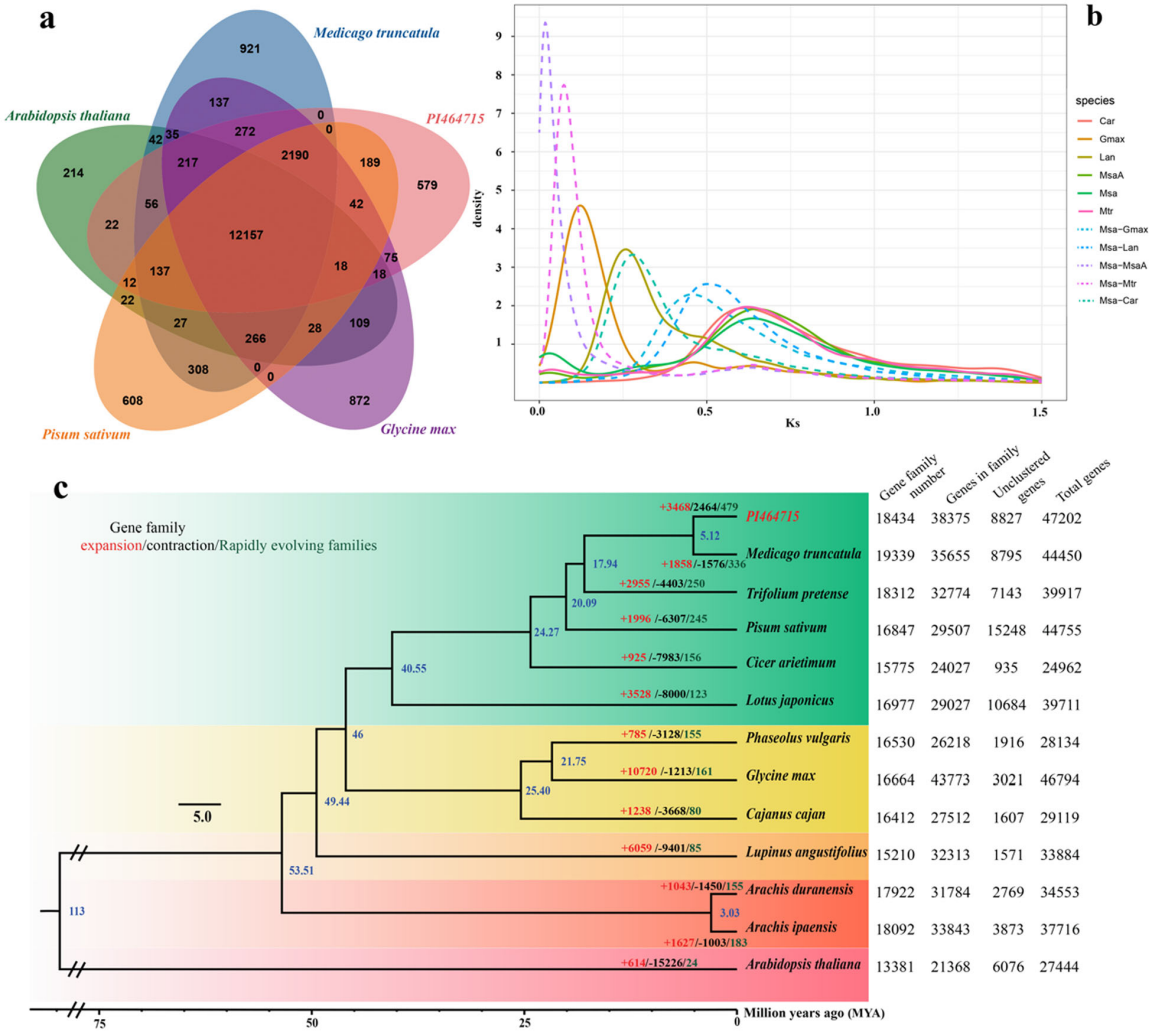
Phylogenetic and evolutionary analyses of the PI464715 genome. **a** Venn diagram of shared orthologous gene families in PI464715, *A. thaliana*, *M. truncatula*, *P. sativum*, and *G. max*. The number of gene families is listed for each component. **b** Distribution of synonymous substitution levels (*K*_s_) of syntenic orthologous (dashed curves) and paralogous genes (solid curves) after evolutionary rate correction. Mtr: *M. truncatula*; Car: *C. arietinum*; Gmax: *G. max*; Lan: *L. angustifolius*; Msa: PI464715. MsaA: *M. sativa* subgenome 1. **c** Phylogenetic tree of 13 plant species and the evolution of gene families. The blue numerical value beside each node shows the estimated divergence time of each node (MYA, million years ago). The numbers of gene-family expansions, contractions and significant (*p*-value ≤ 0.01) gene-family expansion and contraction events are indicated by red, black and green numbers, respectively.

To investigate the genome evolution of PI464715, annotated genes from 11 species of the Leguminosae family (i.e., M. truncatula, Trifolium pertense, Pisum sativum, Cicer arietinum, Lotus japonicus, Phaseolus vulgaris, Glycine max, Cajanus cajan, Lupinus angustifolius, Arachis duranensis, and Arachis ipaensis) and one rosid species (Arabidopsis thaliana) were clustered into gene families. In total, 38,375 PI464715 genes (81.3%) were clustered into 18,434 gene families (Fig. 3c). PI464715 shared a total of 12,157 (65.9%) gene families with the 12 other species and contained 579 (3.1%) unique gene families (Fig. 3a,c). We determined and selected 553 single-copy orthologous genes from these 13 species for subsequent phylogenetic analysis. As expected, PI464715 displayed a close relationship with M. truncatula and phylogenetically diverged from its common ancestor ~5.12 million years ago (Fig. 3c). The phylogenetic relationships among these 13 species were the same as those recovered from a previous study^28^.

Among the 18,434 gene families identified in PI464715, 3468 expanded and 2464 contracted gene families were detected. Compared with its close relative *M. truncatula* (another important species in the genus used as a legume model species), which exhibited 1858 expanded and 1576 contracted gene families, PI464715 had more gene families (Fig. 3c). Furthermore, a higher number of gene families in PI464715 compared with *M. truncatula* (i.e., 479 vs. 336) exhibited significant rapid evolution (family-wide *p*-value ≤ 0.01) (Fig. 2c). The GO enrichment analysis suggested that reproductive processes, such as recognition of pollen (GO:0048544), pollen-pistil interaction (GO:0009875) and pollination (GO:0009856), were enriched in both the contracted and expanded gene families (Supplementary Tables S8 and S9), and these genes may be involved in the transition between self-compatibility in *M. truncatula* and self-incompatibility in PI464715. The GO enrichment analysis of the expanded gene families also suggested multiple response pathways (e.g., response to chemical, response to hormone, response to auxin and response to stimulus), all of which may be related to the adaptation of this species to diverse environments.

### Comparative genomic analyses and genome expansion in PI464715

**Fig. 4:**
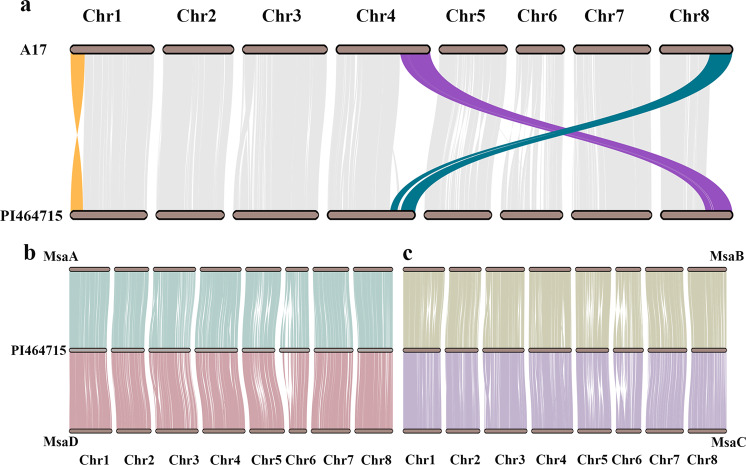
Gene synteny between the *M. truncatula* ecotype Jemalong A17, PI464715, and alfalfa genomes. **a** Synteny comparison of homologous chromosomes between the PI464715 genome and the *M. truncatula* A17 genome. **b** Synteny comparison of homologous chromosomes between the PI464715 genome and the *M. sativa* genome (MsaA: *M. sativa* subgenome 1; MsaD: *M. sativa* subgenome 4). **c** Synteny comparison of homologous chromosomes between the PI464715 genome and the *M. sativa* genome (MsaB: *M. sativa* subgenome 2, MsaC: *M. sativa* subgenome 3).

Synteny analysis was conducted between the PI464715 genome assembly, the four monoploid genomes of alfalfa^19^ and the *M. truncatula* ecotype Jemalong A17 genome v5.0^29^ to explore their evolution. High collinearity was revealed between our genome with all four subgenomes of alfalfa and for five chromosomes between our genome and the A17 genome by visualizing syntenic blocks (Fig. 4). We further detected a pair of large interchromosomal rearrangements between chromosome 4 and chromosome 8 and a large inversion on chromosome 1, as also evident in the dot plots comparing our genome and the A17 genome (Fig. 4a and Supplementary Fig. 4). Such rearrangements and inversions were also found between the genomes of two ecotypes, A17 and R108^30^, but not between the PI464715 and R108 genomes (result not shown). These results indicate that the large interchromosomal rearrangements and inversion may have occurred specifically in A17 after the divergence between *M. truncatula* and *M. sativa*, but this needs further investigation.

Our assembled PI464715 genome is 793 Mb in size, almost two times larger than the genome of *M. truncatula* (420 Mb). We tested whether whole-genome duplication (WGD) events accounted for the genome expansion in PI464715. We selected the genomes of four species (i.e*., M. truncatula*, *C. arietinum*, *G. max*, and *L. angustifolius*) from the Leguminosae family and subgenome A of alfalfa and performed comparative genomic analysis with PI464715 to investigate the WGD events and divergence time between PI464715 and other species, which were evaluated by measuring the synonymous nucleotide substitution rate (*K*_s_) of orthologous gene pairs. All six species displayed a peak *K*_s_ value of 0.62, consistent with the finding of a previous study^31^, and the divergence between PI464715 and the other four species occurred afterwards, suggesting a common whole-genome duplication event for all Papilionoideae species^32,33^. PI464715 and *M. truncatula* experienced no WGD events after their divergence, and the divergence between PI464715 and the diploid ancestor of alfalfa (i.e., represented by one monoploid genome, subgenome A) was the most recent (Fig. 3b).

### Resistance-related (R) genes

**Fig. 5:**
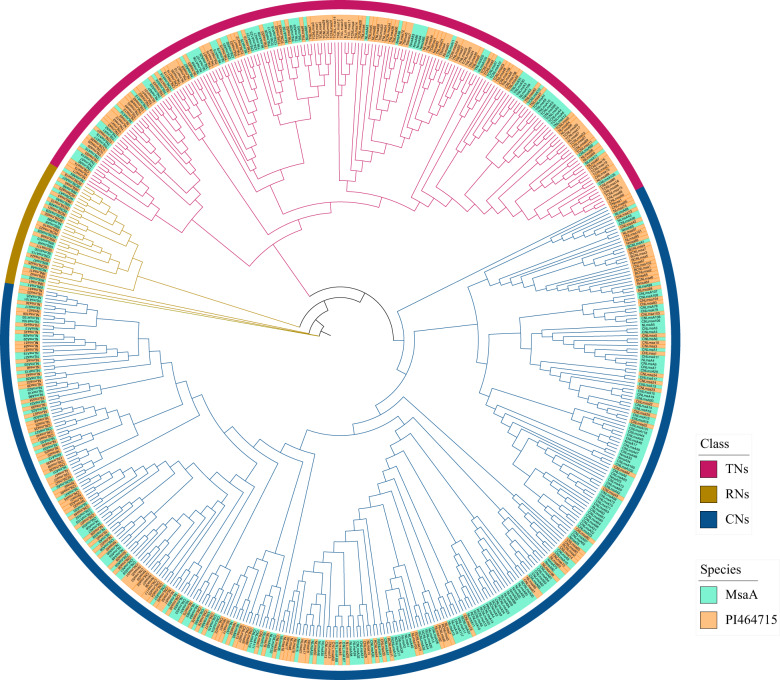
hylogenetic tree of the nucleotide-binding site (NBS) domain R gene-family identified in the PI464715 genome and *M. sativa* A subgenome. (MsaA: *M. sativa* subgenome 1). Class represents the three groups of *R* genes. MsaA: *M. sativa* subgenome 1.

Plant resistance genes (*R* genes) are important gene groups that usually include an NBS (nucleotide-binding site) domain and an LRR (leucine-rich repeat) domain and play a crucial role in plant disease resistance^34^. Based on the types of domains in the N-terminal region, *R* genes belong to three groups: CNL (CC-NBS-LRR), RNL (RPW8-NBS-LRR) and TNL (TIR-NBS-LRR)^35^. In the PI464615 genome, 489 *R* genes were detected, including 117 CNL genes, 58 TNL genes and 11 RNL genes (Supplementary Table S10). The numbers of *R* genes detected in the four monoploid genomes of alfalfa were similar but slightly smaller than those in the PI464615 genome. In total, 1749 *R* genes were detected in the autotetraploid alfalfa genome. Furthermore, PI464615 had ~1.5-fold to ~2.2-fold more TNL genes but fewer CNL genes than the four monoploid genomes of alfalfa. More *R* genes (692) were detected in *M. truncatula*, including 139 CNL genes, 145 TNL genes and 15 RNL genes (Supplementary Table S10). *R* genes with complete domains identified from the PI464715 and *M. truncatula* genomes were selected to construct a phylogenetic tree. The results indicated that these *R* genes were clustered into the RNL, TNL and CNL groups (Fig. 5 and Supplementary Fig. 5).

### Lignin and cellulose biosynthesis-related genes

**Fig. 6:**
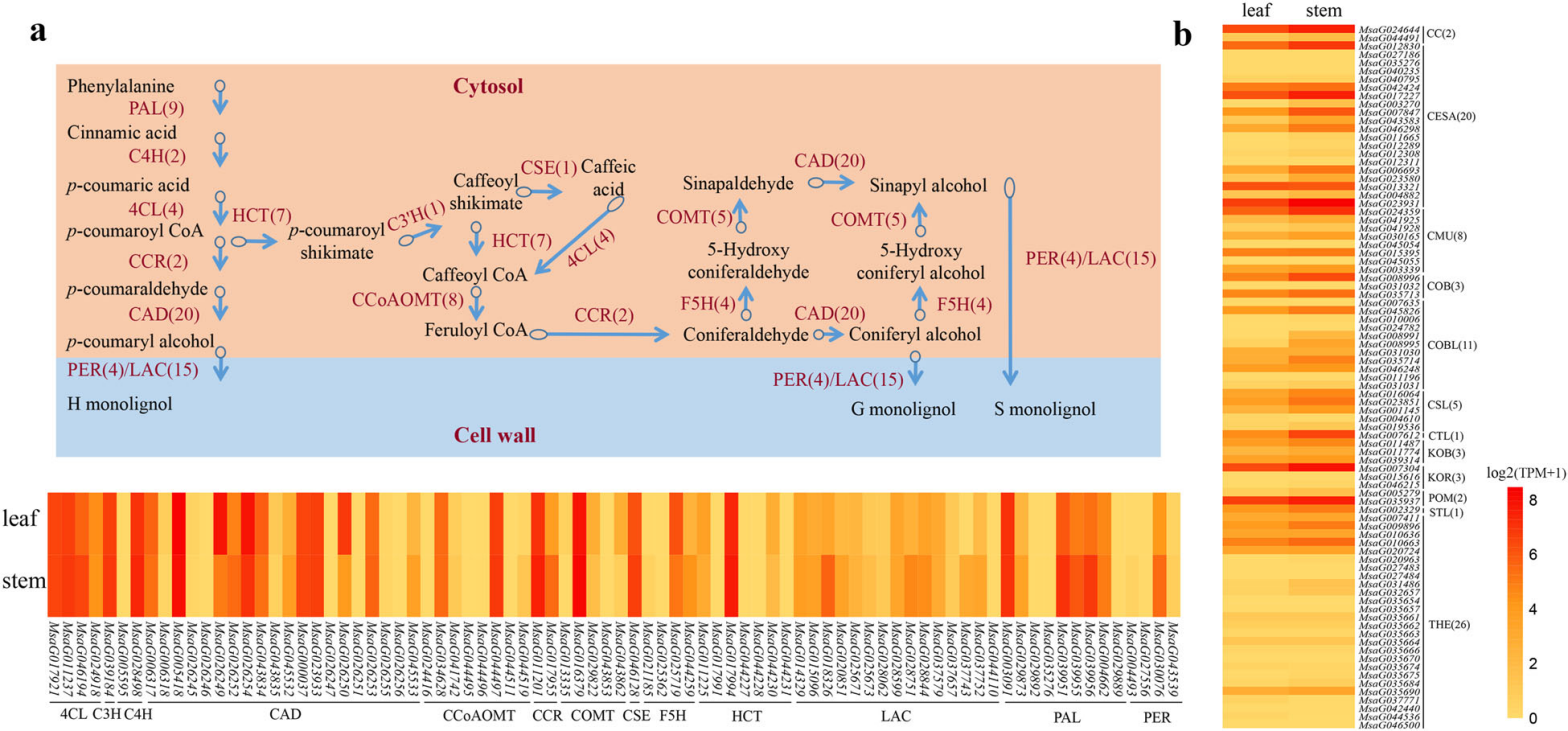
Expression of lignin and cellulose biosynthesis genes in leaf and stem tissues. **a** Simplified diagram of lignin biosynthesis pathways in plants modified from Vanholme et al.^78^ and a heat map showing the expression of lignin biosynthesis-related genes in leaf and stem tissues. Values within brackets indicate the numbers of gene copies corresponding to the catalytic genes in the pathways. The enzymes involved in the lignin biosynthesis pathway are phenylalanine ammonia lyase (PAL), cinnamate 4-hydroxylase (C4H), 4-coumarate-CoA ligase (4CL), cinnamoyl-CoA reductase (CCR), cinnamyl alcohol dehydrogenase (CAD), hydroxycinnamoyl-CoA shikimate/quinate hydroxycinnamoyl transferase (HCT), p-coumaroyl shikimate/quinate 3’-hydroxylase (C3’H), caffeoyl-CoA O-methyltransferase (CCoAOMT), ferulate 5-hydroxylase (F5H), caffeic acid O-methyltransferase (COMT), peroxidase (PER), laccase (LAC) and caffeoyl shikimate esterase (CSE). **b** Heat map showing the expression of cellulose-related genes in leaf and stem tissues. The enzymes involved in cellulose biosynthesis pathways are cellulose synthase (CESA), korrigan (KOR), chitinase-like (CTL), cobra (COB), cobra-like (COBL), kobito (KOB), stello (STL), cellulose synthase-microtubule uncoupling protein (CMU), companion of cellulose synthase (CC), theseus (THE), cellulose synthase interacting (POM), and tracheary element differentiation-related (TED).

The content of lignin and cellulose is one of the important factors affecting alfalfa quality as an animal feed^36^, and reducing the lignin content in alfalfa can improve digestibility and, correspondingly, animal performance^37^. Based on a BLASTp homology search and Pfam analysis, we identified a total of 82 putative lignin biosynthesis-related genes and 85 putative cellulose biosynthesis-related genes (Fig. 6). These genes were unevenly distributed on the eight chromosomes (Supplementary Fig. 6). Hierarchical cluster analysis of transcriptomic data showed clustering of the three repeats for the leaf or stem (Supplementary Fig. 7). Transcriptomic analysis revealed that the expression patterns of these identified genes involved in the lignin and cellulose biosynthesis pathways in leaf and stem tissues were similar, but the expression levels were slightly higher in stem than in leaf tissue (Fig. 6), which is consistent with the fact that the lignin content is higher in stem than in leaf tissue. We also found that the expression levels of multiple gene copies for each gene were different. For example, among the seven gene copies that putatively encode the enzyme HCT, *MsaG017994* had the highest expression level, which was 13–250 times higher than that of other gene copies (Fig. 6a). Knowing the relative expression levels of different gene copies can be useful when conducting targeted downregulation of enzymes for forage quality improvement by reducing lignin content, for example^36,38,39^.

## Discussion

*Medicago* includes economically important forage crops, such as alfalfa (*M. sativa*) and “Jinhuacai” (*M. polymorpha*), in addition to a model organism (*M. truncatula*) in plant biology. Despite the importance of the genus, genomic resources are relatively scarce, and genome sequences are available only for *M. truncatula* and alfalfa, which largely slows down progress towards understanding the genome evolution and genetic code underlying molecular breeding for major crops in this genus. Here, we describe a chromosome-scale assembly of the *M. sativa* ssp. *caerulea* genome (i.e., the diploid progenitor of autotetraploid alfalfa) obtained by a combination of data from the Illumina, Nanopore and Hi-C platforms. The genome assembly was 793 Mb in length, and >98.5% of the assembled genome was placed on eight chromosomes (Table 1 and Supplementary Table S5). The BUSCO assessment revealed 97.7% complete genes in the assembled genome, which represents a more contiguous and higher-quality genome assembly than that recently published for alfalfa^19^. Our results further reveal that Nanopore long reads with the aid of Hi-C data can be adopted to accurately assemble a highly heterozygous and repetitive genome^40^.

The PI464615 genome (793 Mb) is approximately twofold larger than that of the closely related species *M. truncatula* (420 Mb)^29^. Several factors, including transposable elements (TEs) and whole-genome duplication (WGD), have been proposed to account for variation in genome size^41,42^. Recent analyses have shown that WGD or polyploidization seems to have occurred during the evolutionary histories of most plant species, such as the γ event^43^ that occurred ~140–150 Myr ago^44^ and is shared by all eudicots. After the γ-event, some species experienced no WGD events, such as grape and coffee, whereas other species, such as *M. truncatula*, kiwifruit and *Asparagus setaceus*, may have undergone one or two additional rounds of WGD^29,45,46^. All Papilionoideae within the Leguminosae family share a common WGD event, after which most species experienced no WGD events, except for *G. max* and *L. angustifolius*^47–49^. Our results from *K*_s_ distribution analysis reveal that both the PI464615 and *M. truncatula* genomes have only one peak, which precedes the divergence of the two species and is consistent with the ancestral Papilionoideae WGD event. The proliferation of TEs is another factor accounting for genome expansion. In this study, we identified ~440 Mb TEs, constituting 55.5% of the assembled PI464615 genome, which is ~234 Mb larger than the total length of TEs (~206 Mb)^29^ in the *M. truncatula* genome. Therefore, the proliferation of TEs rather than WGD and the presence of more expanded gene families than in *M. truncatula* resulted in genome expansion in PI464615.

In summary, we report a high-quality chromosome-level reference genome for *M. sativa* ssp. *caerulea* (voucher PI464715). We assembled a 793 Mb genome and annotated 47,202 protein-coding genes. We also identified resistance genes in the PI464715 genome and in each of the four monoploid genomes of alfalfa, which may provide a genetic basis for understanding the gain of resistance-related traits in alfalfa. We further identified 82 and 85 candidate genes that may be involved in the lignin and cellulose biosynthesis pathways, respectively, and described the expression profiles of these genes in leaf and stem tissues. Such information will be very useful for improving alfalfa quality in the future, for example, by the downregulation of targeted enzymes^36,38,39^ or through gene editing to decrease lignin content. The available genome sequence for the direct progenitor of autotetraploid alfalfa is an important complement to the alfalfa genome and holds great promise for further understanding fundamental aspects of genomic architecture and improving molecular breeding strategies in alfalfa. The genomic resource is also highly valuable for evolutionary studies in related species.

## Material and methods

### Plant materials, DNA extraction, and estimation of genome size

Seeds of *M. sativa* spp*. caerule*a voucher PI464715 were obtained from the National Plant Germplasm System (NPGS) of the United States Department of Agriculture (USDA) and planted in a greenhouse. Fresh leaves of a growing plant cultivated in a greenhouse were used to extract genomic DNA using a DNA Secure Plant Kit (Tiangen Biotech, Co., Ltd., Beijing, China). Paired-end libraries with insert sizes of 270 bp were constructed, and the Illumina HiSeq X Ten platform was used to generate Illumina short reads, which were first used to estimate genome size. We generated ~81.5 Gb of reads and determined the abundance of 17-K-mers in the generated Illumina data using Kmerfreq^50^. K-mer curve fitting was also performed under different gradient combinations of heterozygosity to estimate the heterozygosity of the genome.

### Genome sequencing and assembly

Total genomic DNA was fractionated into 10–50 kb fragments with BluePippin, which was used to construct the libraries following the Nanopore library construction protocol. The generated libraries were then submitted for sequencing at the Nextomics Biosciences Company (Wuhan, China) using the GridION X5 sequencer platform (Oxford Nanopore Technologies, UK). The quality-controlled reads were used for assembly with the software Nextdenovo v. 2.3.0^51^ following three steps. First, the NextCorrect module was applied to correct sequencing errors. Second, a preliminary assembly was generated based on the NextGraph module, which resulted in a genome size of 1345.8 Mb, with a contig number of 1154 and contig N50 of 2.8 Mb. Then, we polished the preliminary assembly using the Nextpolish v. 1.2.4^24^ module. At this stage, Nanopore long reads and Illumina short reads were used repetitively three times for genome correction. Finally, allelic haplotigs were removed using purge_haplotigs^25^ software to obtain the final genome sequence. BUSCO v. 2.0^26^, with 1,350 genes from Embryophyta odb10, was used to evaluate the completeness and accuracy of the assembled genome.

### Chromosome-scale assembly with Hi-C data

Approximately 2 g of fresh leaves collected from the same PI464715 accession was used for Hi-C sequencing. Hi-C libraries were constructed following Miele et al.^52^ with chromatin extraction; digestion; and DNA ligation, purification and fragmentation. Hi-C sequencing was performed using the Illumina HiSeq X Ten platform (Illumina, CA, USA). A preliminary assembly was carried out to correct errors in contigs by splitting contigs into 100 kb segments on average. BWA v. 0.7.17^53^ was used to map the Hi-C data to these segments. The uniquely mapped Hi-C data were retained, clustered, ordered and placed onto the eight pseudochromosomes using LACHESIS^28^. A heat map of the interaction matrix of all pseudochromosomes was plotted with a resolution of 100 kb.

### Repetitive sequence and gene annotation

Repetitive elements in the PI464715 genome assembly were identified based on a combination of homology-based and de novo approaches at both the protein and DNA levels. First, TRF v. 4.0.7^54^ was applied to identify the tandem repeats in the genome assembly. Then, TEs were identified using RepeatMasker v. 4.1.0^55^ and RepeatProteinMask (http://www.repeatmasker.org/) with Repbase^51^ as the query library. Next, RepeatModeler v. 5.8.8^56^ (http://www.repeatmaskerorg/) was used to construct a de novo repeat library for the identification of TEs that were not found in the Repbase library.

We predicted protein-coding genes using a combination of de novo prediction, homology-based prediction and transcriptome-based prediction. Augustus v. 3.3.2^57^, GlimmerHMM v. 3.0.4^58^, Geneid v. 1.4.5^59^, and Genscan^60^ software were used for de novo prediction. GeMoMa v. 1.3.1^61^ was used for homology prediction, with protein sequences from *M. truncatula, C. arietinum, G. max, P. vulgaris, P. persica* and *A. thaliana*. For transcriptome-based predictions, we first sequenced the RNA library generated from mixed stem, leaf and flower tissues, and the RNA-seq reads were assembled into transcripts using Trinity v. 2.1.1^62^ with default parameters. In addition, we mapped all the RNA-seq reads to the final assembled genome by PASA v. 2.1.0^63^ to assess genome assembly quality. To annotate the noncoding RNAs, tRNAscan-SE v. 1.3.1^64^ was applied for identifying tRNA genes with eukaryotic parameters. BLAST^65^ was applied to search the rRNA sequences in the PI464715 genome assembly with default parameters. MiRNA and snRNA were identified using INFERNAL v. 1.1^66^ software based on covariance models deposited in the Rfam v. 13.0^67^ database.

Gene functions were annotated by performing BLAST^65^ (*E-*value ≤ 1e^−5^) searches against four protein databases, i.e., SwissProt, KOG, NR, and KEGG. The InterPro database with BLAST or InterProScan v. 4.8^68^ was used to annotate the functions of protein-coding genes. UniProt and GO annotations were assigned for each protein based on the results of alignment.

### Gene families and phylogenetic analysis

We used OrthoFinder v. 2.2.7^69^ to identify the orthologous groups among 12 Leguminosae species (PI464715, *M. truncatula*, *T. pretense*, *P. sativum*, *C. arietimum*, *L. japonicus*, *P. vulgaris*, *G. max*, *C. cajan*, *L. angustifolius*, *A. duranensis*, *A. ipaensis*) and one rosid species (*A. thaliana*). We then extracted the single-copy orthologous genes from the orthologous clustering results. We used CAFÉ v. 2.2^70^ software to identify the expanded and contracted gene families in the 13 species, which were further subjected to GO enrichment analysis. For phylogenetic analysis, we first used MAFFT to perform multiple alignments of protein sequences of single-copy orthologous genes with default parameters. Then, the protein sequence alignments were converted into codon alignments. Second, Gblocks v. 0.91^71^ was used to delete regions with poor alignment or large differences in the results of multiple sequence alignments. Finally, the codon alignment results of all single-copy orthologs were connected to form a supergene for phylogenetic analysis. RAxML v. 8.2.0^72^ was used to construct the phylogenetic tree. We calculated the average substitution rate along each branch and estimated species divergence time using r8s v. 1.81^73^.

### Gene collinearity and *K*_s_ analysis

Syntenic blocks between PI464715 and the four monoploid genomes of alfalfa and *M. truncatula* ecotype Jemalong A17 were detected using MCScanX^74^. The number of synonymous substitutions per synonymous site (*K*_s_) on each branch was estimated using the codeml program in the PAML v. 4.0 package^75^, and the median *K*_s_ value was representative of the collinear blocks.

### Identification of resistance (*R*) genes

We used both BLAST searches and the hidden Markov model (HMM) to obtain *R* genes in the PI464715 genome, the four monoploid genomes of alfalfa and *M. truncatula* genomes. All of the protein sequences annotated in these genomes were first searched by using the HMM profile of the NB-ARC domain (Pfam no. PF00931) in a hmmscan subprocess of HMMER 3.2.1 (http://hmmer.org/). We used BLASTp to search the amino acid sequences of the NB-ARC domain against all annotated protein sequences in each genome. We merged all hits obtained from both analyses and removed the redundant hits. The sequences were further subjected to Pfam analysis and coiled-coil (CC) analysis to identify TIR, LRR, RPW8, zf-BED and CC domains. The method was similar to that used in a previous study^76^. We used paircoil2^77^ (the threshold value was set to 0.025) and coils software to identify CC domains.

### Identification and expression of lignin and cellulose biosynthesis genes

To identify the genes involved in the lignin and cellulose biosynthesis pathways in PI464715, we used the genes listed in the diagram of lignin biosynthesis pathways in plants by Vanholme et al.^78^ and cellulose biosynthesis genes identified in the *A. thaliana* database as references^79^. Then, the BLASTp algorithm and Pfam analysis were used to search our genome for homologs. The locations of all identified lignin and cellulose biosynthesis genes were marked on the eight chromosomes by MapChart v. 2.32 software^80^.

To examine the expression of these lignin and cellulose biosynthesis-related genes, we carried out RNA sequencing of two tissues (i.e., leaf and stem, each with three replicates) through 2 × 151-bp paired-end libraries using an Illumina HiSeq 4000. Leaf and stem tissues were obtained from a voucher PI464715 plant. Raw Illumina reads of low quality (when the percentage of low-quality bases was over 50% in a read) and with unknown bases (>10%) were filtered out to obtain clean reads. Then, the clean reads were mapped to the genome assembly using HISAT2 v. 2.0.4^81^ with default parameters. Read alignments for transcripts in each sample were extracted using StringTie v. 1.2.3^82^. The expression level of each gene was measured by transcripts per million (TPM) values estimated in StringTie.

## Supplementary information

Supporting information

## Data Availability

The whole-genome sequence data (including Illumina short reads, Nanopore long reads and Hi-C interaction reads), the final assembled genome and the transcriptomes of different tissues used in this study have been deposited in the NCBI database under BioProject ID PRJNA657344. The genome annotation information has been uploaded to Figshare.
