# Supplementary material for: A chromosome-scale genome assembly of a diploid alfalfa, the progenitor of autotetraploid alfalfa
Source: Hortic Res. 2020 Dec 1;7:194. doi: 10.1038/s41438-020-00417-7 (PMC7705661; doi:10.1038/s41438-020-00417-7)
Supplement: Supplementary file 1 — Supporting information [file 41438_2020_417_MOESM1_ESM.doc]

**Supplementary Information**

**Supplementary Figures**

**
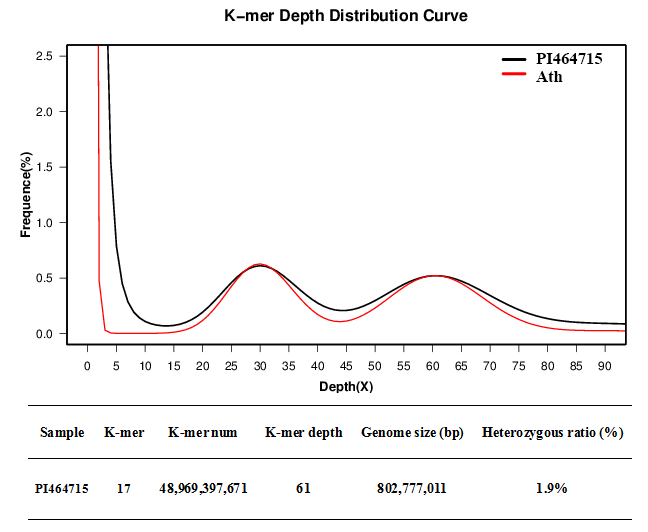
**

**Supplementary Fig. 1** **K-mer analysis of the PI464715 and *Arabidopsis thaliana* genomes by using K-mer = 17.**

Distribution of 17-mer depth calculated from filtered reads of a PCR-free library. The genome size is estimated using the following formula: Genome size = K-mer Number / Peak Depth.


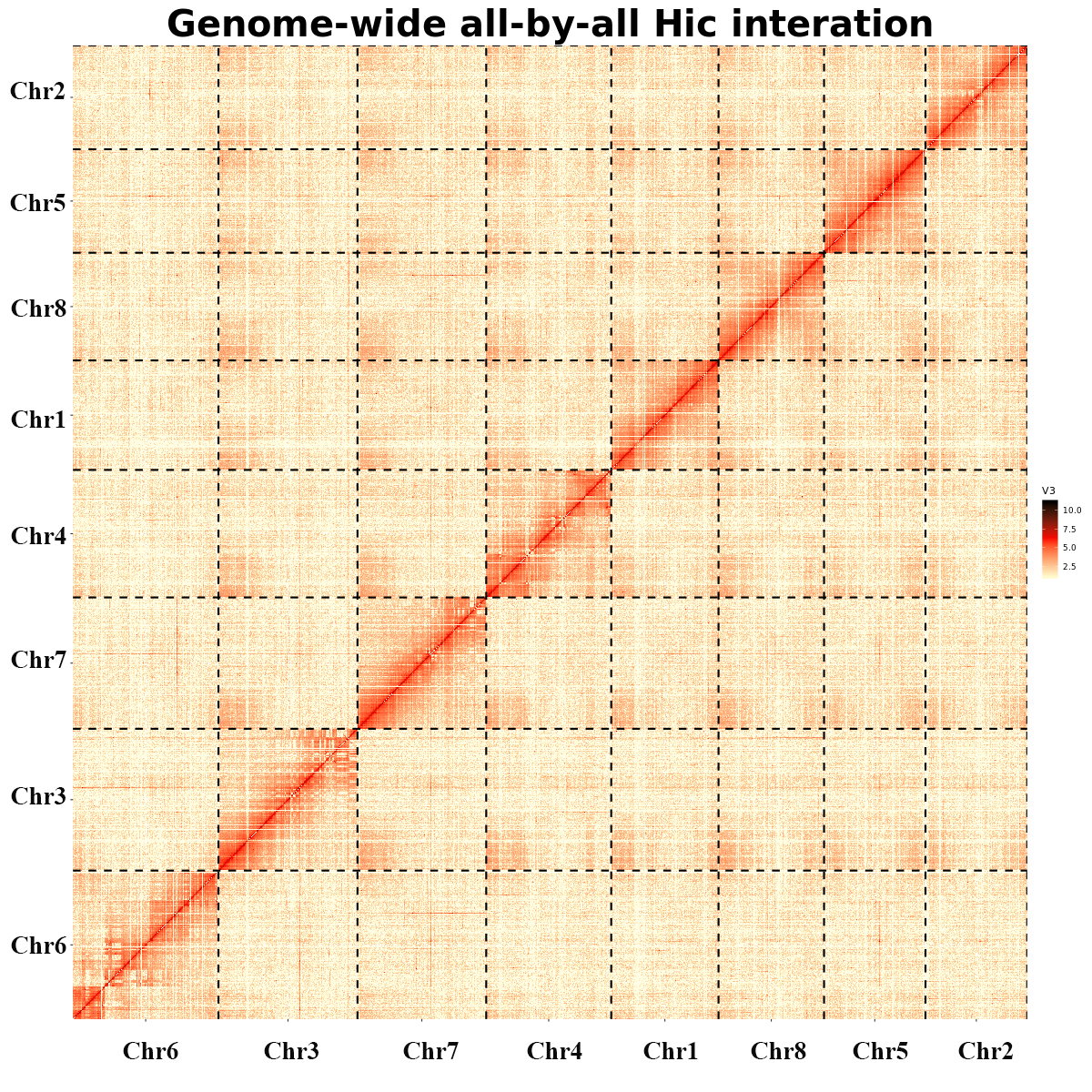


**Supplementary Fig. 2 Intensity signal heat map of the Hi-C chromosome.**

**
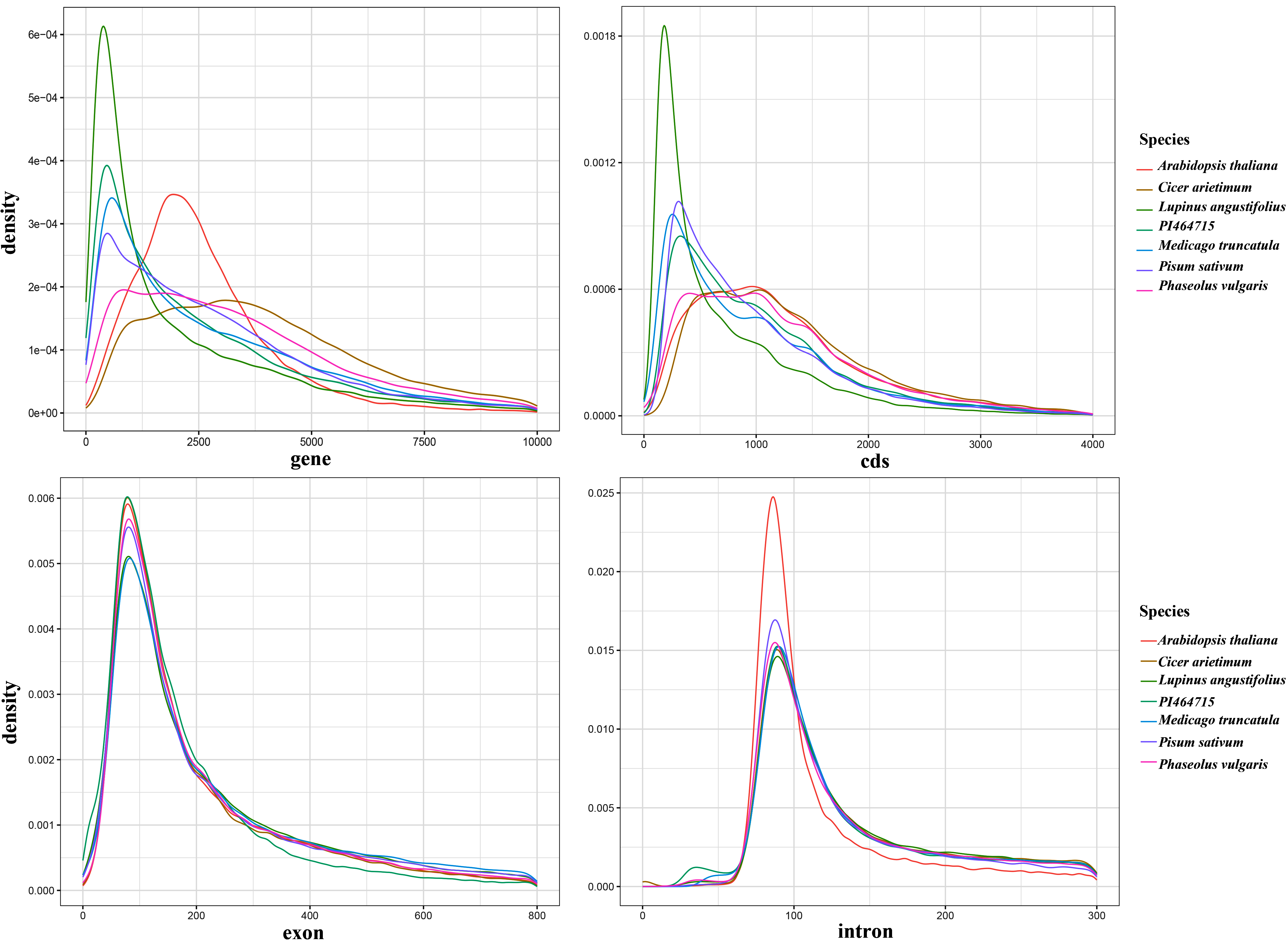
**

**Supplementary Fig. 3 Gene structure prediction statistical results of the PI464715 genome compared with genetic elements of related species.**


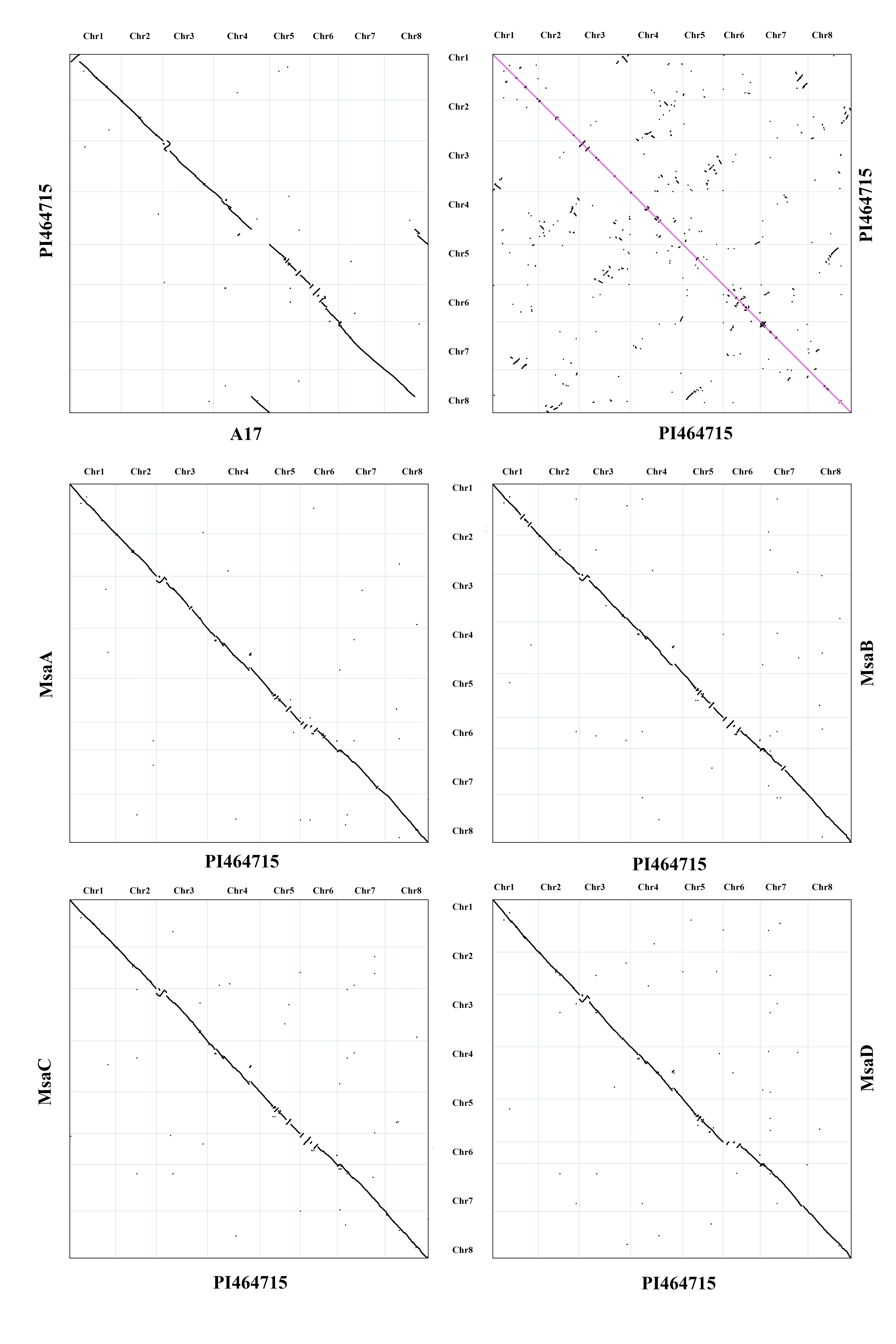
**Supplementary Fig. 4 Dot plot showing gene synteny between the PI464715, *M. truncatula* A17 genome and *M. sativa* (alfalfa) genome (MsaA: *M. sativa* subgenome 1; MsaB: *M. sativa* subgenome 2; MsaC: *M. sativa* subgenome 3; MsaD: *M. sativa* subgenome 4).**

**
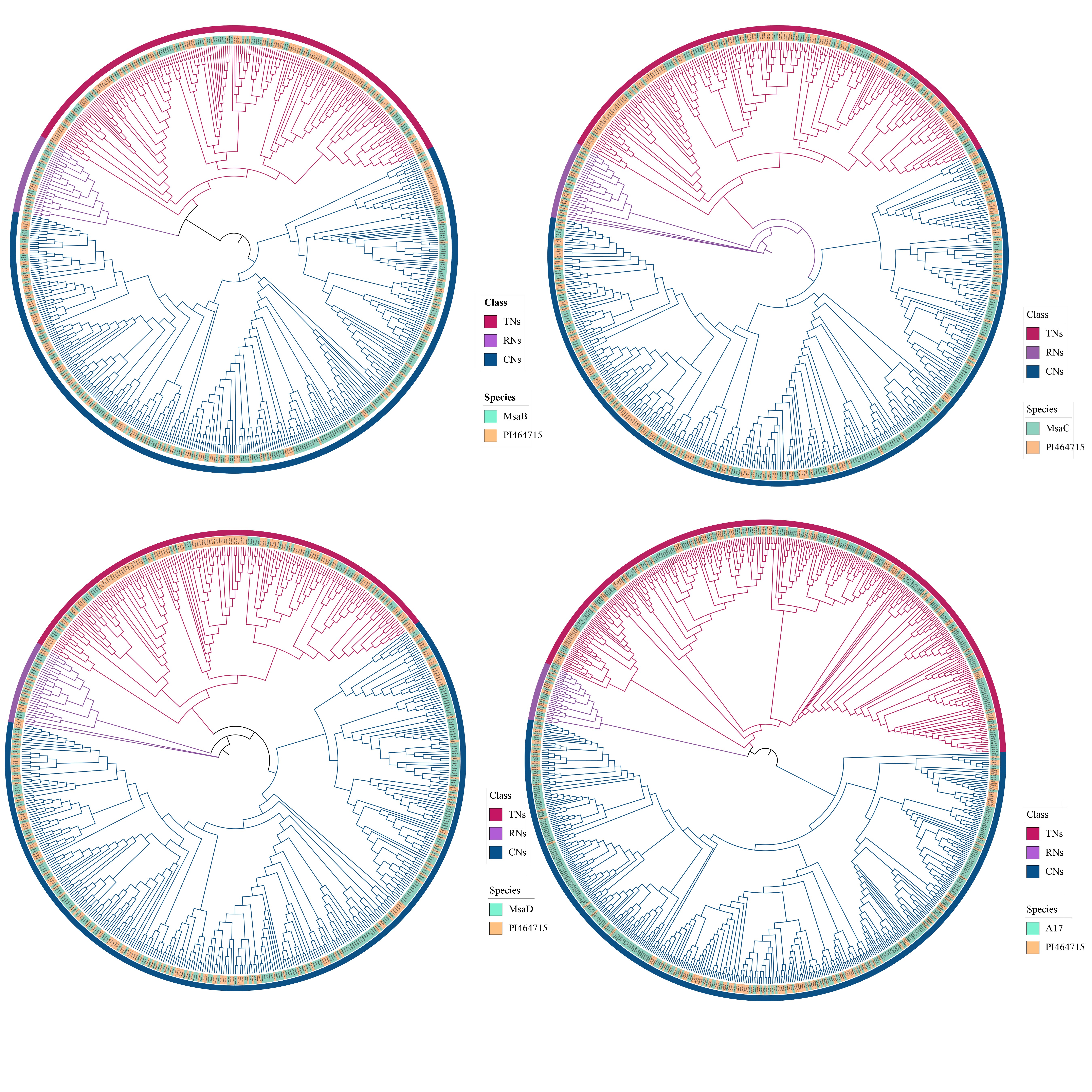
**

**Supplementary Fig. 5 Phylogenetic tree of the nucleotide-binding site (NBS) domain *R* genes in *M. truncatula* ecotype Jemalong A17, PI464715 and *M. sativa* genomes.**

Blue branches indicate the coiled-coil-NBS-LRR (CNL) genes, red branches indicate TIR-NBS-LRR (TNL) and purple branch indicates the RPW8-NBS-LRR (RNL). **a:** Phylogenetic tree of *R* genes identified from PI464715 (orange) genome and *M. sativa* subgenome 2 (green). **b:** Phylogenetic tree of *R* genes identified from PI464715 (orange) genome and *M. sativa* subgenome 3 (green). **c:** Phylogenetic tree of *R* genes identified from PI464715 (orange) genome and *M. sativa* subgenome 4 (green). **d:** Phylogenetic tree of *R* genes identified from A17 (green) and PI464715 (orange) genomes.

**
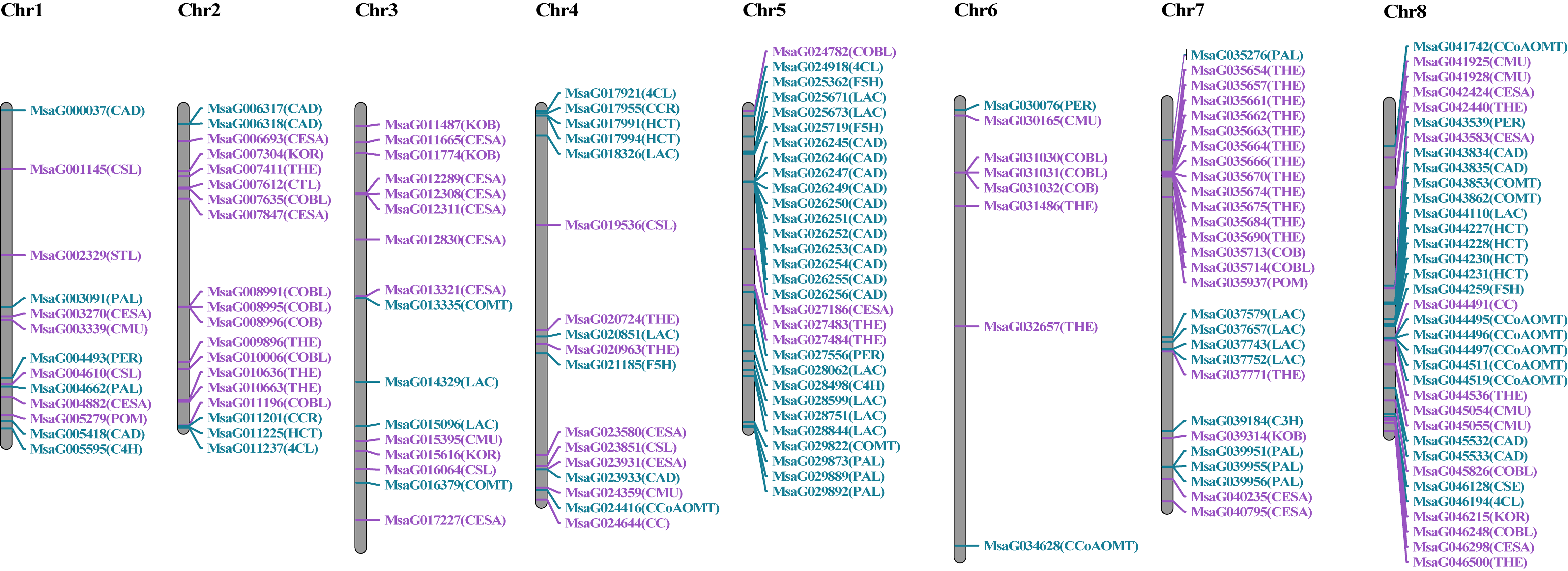
**

**Supplementary Fig. 6 Chromosomal locations of lignin and cellulose biosynthesis related genes in PI464715.**

Green and purple represent lignin and cellulose related genes, respectively.

**
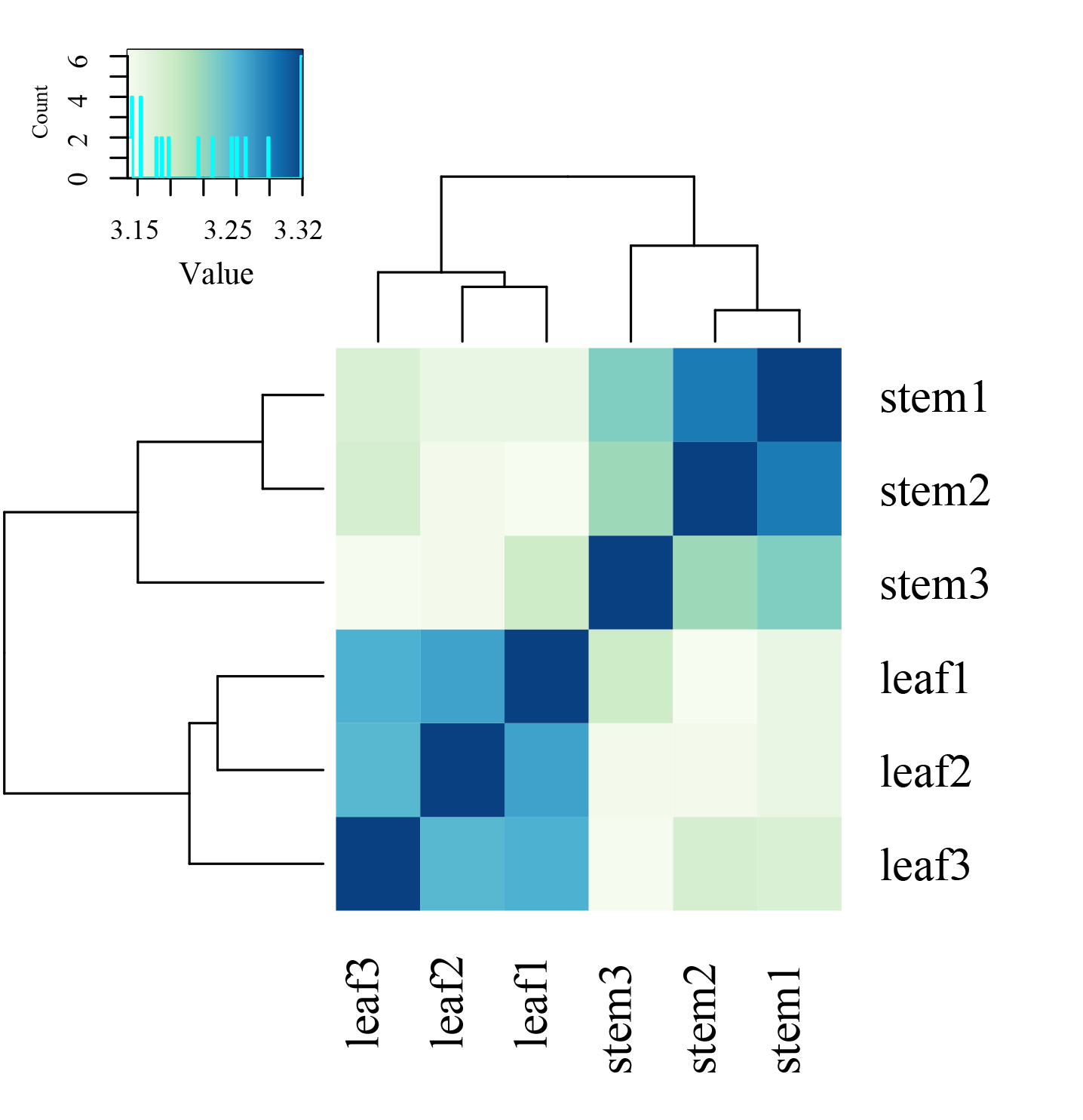
**

**Supplementary Fig. 7 Hierarchical cluster analysis of gene expression in leaf and stem tissues.**

**Supplementary Tables**

# Supplementary Table S1 Summary of DNA sequencing data.

| **Read type** | **Number of clean reads / subreads** | **Insert size (bp)** | **Total data (G**b) | **Read length / Mean subread length (N50) (bp)** | **Sequence coverage**^1^ (X) |
| --- | --- | --- | --- | --- | --- |
| **Illumina pair-end reads** | 587,127,922 | 270 | 81.5 | 2 x 150 | 102 |
| **Hi-C pair-end reads** | 751,799,402 | 300 | 224 | 2 x 150 | 279 |
| **Nanopore long reads** | 5,899,924 | - | 116.5 | 19,700 (27,900) | 145 |

^1^Depth was calculated under the estimate of a genome size of 802 Mb.

**Supplementary Table S2 Genome assembly completeness evaluation with 1,375 BUSCO groups.**

|  | **Genome** | |
| --- | --- | --- |
| **Type** | **Number** | **Percent (%)** |
| Complete BUSCOs* (C) | 1,343 | 97.7 |
| Complete and single-copy BUSCOs (S) | 1,224 | 89.0 |
| Complete and duplicated BUSCOs (D) | 119 | 8.7 |
| Fragmented BUSCOs (F) | 8 | 0.6 |
| Missing BUSCOs (M) | 24 | 1.7 |
| Total BUSCO groups searched | 1,375 | - |

**Supplementary Table S3** Chromosome length by Hi-C assembly.

| **Group** | **Sequence Length (bp)** | **Scaf Num** | **Chromosome** | **Size (Mb）** | **GC%** | **Gene** |
| --- | --- | --- | --- | --- | --- | --- |
| Lachesis Group 5 | 87,192,649 | 51 | Chr1 | 87.19 | 33.87 | 5,965 |
| Lachesis Group 8 | 83,243,105 | 36 | Chr2 | 83.24 | 33.98 | 5,285 |
| Lachesis Group 2 | 114,224,159 | 41 | Chr3 | 114.22 | 34.41 | 6,625 |
| Lachesis Group 4 | 102,487,784 | 40 | Chr4 | 102.49 | 34.15 | 6,835 |
| Lachesis Group 7 | 83,513,093 | 35 | Chr5 | 83.51 | 34.02 | 5,225 |
| Lachesis Group 1 | 118,420,570 | 56 | Chr6 | 118.42 | 34.73 | 4,819 |
| Lachesis Group 3 | 105,800,569 | 36 | Chr7 | 105.80 | 34.36 | 6,218 |
| Lachesis Group 6 | 86,183,107 | 42 | Chr8 | 86.18 | 33.90 | 5,607 |
| Un | 12,160,362 | 17 | - | 12.16 | 33.84 | 623 |
| Total | 793,225,398 | 355 | - | 793.2 | 34.21 | 47,202 |

Supplementary Table S4 Summary of RNA sequencing data.

| **Tissue** | **Read type** | **Number of reads** | **Total data (G**b) | **Read length (bp)** | **Mapping rate (%)** |
| --- | --- | --- | --- | --- | --- |
| Leaf+Stem+Flower | Illumina paired-end | 23,801,370 | 7.14 | 2 x 150 | 98.31 |

**Supplementary Table** S5 Statistics of reads mapping rate of PI464715 genome assembly.

| **Library** | **Total reads** | **Mapped reads** | **Mapped rate (%)** | **Properly mapped reads** | **Properly_mapped rate (%)** |
| --- | --- | --- | --- | --- | --- |
| 270bp | 590,140,261 | 588,610,240 | 99.74 | 566,163,724 | 96.38 |

**Supplementary Table S6 Gene set completeness evaluation with 1,375 BUSCO groups**

|  | **Gene** | |
| --- | --- | --- |
| **Type** | **Number** | **Percent (%)** |
| Complete BUSCOs* (C) | 1,333 | 97.0 |
| Complete and single-copy BUSCOs (S) | 1,167 | 84.9 |
| Complete and duplicated BUSCOs (D) | 166 | 12.1 |
| Fragmented BUSCOs (F) | 22 | 1.6 |
| Missing BUSCOs (M) | 20 | 1.4 |
| Total BUSCO groups searched | 1,375 | - |

**Supplementary Table S7** **The statistical results of functional annotation.**

|  | **Numbers of matching genes** | **Percent of annotated genes (%)** |
| --- | --- | --- |
| Total | 47,203 | 100 |
| InterPro | 35,913 | 76.08 |
| KEGG | 11,021 | 23.35 |
| Swissprot | 31,536 | 66.81 |
| KOG | 32,934 | 69.77 |
| NR | 43,158 | 91.43 |
| Annotated^1^ | 43,669 | 92.51 |
| Unannotated | 3,534 | 7.49 |

^1^At least one match in either of database above.

**Supplemental Table S8 GO enrichment analysis of contracted gene families in the PI464715** genome.

| **GO ID** | **GO Name** | **GO Category** | **P-Value** | **Number of genes** |
| --- | --- | --- | --- | --- |
| GO:0015074 | DNA integration | Biological process | 1.33E-15 | 10 |
| GO:0006468 | protein phosphorylation | Biological process | 1.88E-07 | 25 |
| GO:0016310 | phosphorylation | Biological process | 7.81E-07 | 25 |
| GO:0006259 | DNA metabolic process | Biological process | 2.37E-06 | 10 |
| GO:0006796 | phosphate-containing compound metabolic process | Biological process | 7.02E-06 | 26 |
| GO:0006793 | phosphorus metabolic process | Biological process | 7.16E-06 | 26 |
| GO:0006464 | cellular protein modification process | Biological process | 1.32E-05 | 25 |
| GO:0036211 | protein modification process | Biological process | 1.32E-05 | 25 |
| GO:0043412 | macromolecule modification | Biological process | 3.10E-05 | 25 |
| GO:0044260 | cellular macromolecule metabolic process | Biological process | 3.21E-05 | 42 |
| GO:0008037 | cell recognition | Biological process | 5.61E-04 | 5 |
| GO:0009856 | pollination | Biological process | 5.61E-04 | 5 |
| GO:0009875 | pollen-pistil interaction | Biological process | 5.61E-04 | 5 |
| GO:0048544 | recognition of pollen | Biological process | 5.61E-04 | 5 |
| GO:0044706 | multi-multicellular organism process | Biological process | 5.61E-04 | 5 |
| GO:0000003 | reproduction | Biological process | 0.001198773 | 5 |
| GO:0022414 | reproductive process | Biological process | 0.001198773 | 5 |
| GO:1901363 | heterocyclic compound binding | Molecular function | 5.32E-13 | 96 |
| GO:0097159 | organic cyclic compound binding | Molecular function | 5.32E-13 | 96 |
| GO:0043531 | ADP binding | Molecular function | 1.30E-12 | 21 |
| GO:0032559 | adenyl ribonucleotide binding | Molecular function | 1.46E-10 | 51 |
| GO:0030554 | adenyl nucleotide binding | Molecular function | 1.55E-10 | 51 |
| GO:0043167 | ion binding | Molecular function | 8.78E-10 | 75 |
| GO:0005488 | binding | Molecular function | 9.50E-10 | 126 |
| GO:0032555 | purine ribonucleotide binding | Molecular function | 1.98E-09 | 52 |
| GO:0017076 | purine nucleotide binding | Molecular function | 2.24E-09 | 52 |
| GO:0032553 | ribonucleotide binding | Molecular function | 3.02E-09 | 52 |
| GO:0097367 | carbohydrate derivative binding | Molecular function | 3.76E-09 | 52 |
| GO:0000166 | nucleotide binding | Molecular function | 2.12E-08 | 53 |
| GO:1901265 | nucleoside phosphate binding | Molecular function | 2.12E-08 | 53 |
| GO:0036094 | small molecule binding | Molecular function | 1.02E-07 | 53 |
| GO:0043168 | anion binding | Molecular function | 1.07E-07 | 52 |
| GO:0003899 | DNA-directed 5'-3' RNA polymerase activity | Molecular function | 1.68E-07 | 7 |
| GO:0016772 | transferase activity, transferring phosphorus-containing groups | Molecular function | 1.70E-07 | 33 |
| GO:0034062 | 5'-3' RNA polymerase activity | Molecular function | 8.07E-07 | 7 |
| GO:0097747 | RNA polymerase activity | Molecular function | 8.07E-07 | 7 |
| GO:0005506 | iron ion binding | Molecular function | 8.64E-06 | 14 |
| GO:0016705 | oxidoreductase activity, acting on paired donors, with incorporation or reduction of molecular oxygen | Molecular function | 1.09E-05 | 14 |
| GO:0004672 | protein kinase activity | Molecular function | 1.14E-05 | 25 |
| GO:0016301 | kinase activity | Molecular function | 2.74E-05 | 26 |
| GO:0016773 | phosphotransferase activity, alcohol group as acceptor | Molecular function | 5.19E-05 | 25 |
| GO:0020037 | heme binding | Molecular function | 6.80E-05 | 14 |
| GO:0046906 | tetrapyrrole binding | Molecular function | 7.15E-05 | 14 |
| GO:0016779 | nucleotidyltransferase activity | Molecular function | 1.69E-04 | 7 |
| GO:0046914 | transition metal ion binding | Molecular function | 2.72E-04 | 23 |
| GO:0140098 | catalytic activity, acting on RNA | Molecular function | 6.22E-04 | 11 |
| GO:0005524 | ATP binding | Molecular function | 0.002283306 | 30 |

**Supplemental Table S9 GO enrichment analysis of expanded gene families in the PI464715** genome.

| **GO ID** | **GO Name** | **GO Category** | **P-Value** | **Number of genes** |
| --- | --- | --- | --- | --- |
| GO:0042221 | response to chemical | Biological process | 0 | 110 |
| GO:0006508 | proteolysis | Biological process | 0 | 160 |
| GO:0009719 | response to endogenous stimulus | Biological process | 0 | 110 |
| GO:0009725 | response to hormone | Biological process | 0 | 110 |
| GO:0050896 | response to stimulus | Biological process | 0 | 145 |
| GO:0032501 | multicellular organismal process | Biological process | 0 | 47 |
| GO:0010033 | response to organic substance | Biological process | 0 | 110 |
| GO:0009733 | response to auxin | Biological process | 1.11E-16 | 110 |
| GO:0006869 | lipid transport | Biological process | 3.33E-16 | 36 |
| GO:0010876 | lipid localization | Biological process | 9.99E-16 | 36 |
| GO:0007275 | multicellular organism development | Biological process | 2.46E-11 | 20 |
| GO:0048856 | anatomical structure development | Biological process | 2.49E-10 | 20 |
| GO:0032502 | developmental process | Biological process | 2.49E-10 | 20 |
| GO:0048544 | recognition of pollen | Biological process | 9.15E-09 | 27 |
| GO:0009856 | pollination | Biological process | 9.15E-09 | 27 |
| GO:0009875 | pollen-pistil interaction | Biological process | 9.15E-09 | 27 |
| GO:0044706 | multi-multicellular organism process | Biological process | 9.15E-09 | 27 |
| GO:0008037 | cell recognition | Biological process | 9.15E-09 | 27 |
| GO:0006414 | translational elongation | Biological process | 1.11E-08 | 21 |
| GO:0015074 | DNA integration | Biological process | 5.65E-08 | 13 |
| GO:0019538 | protein metabolic process | Biological process | 2.26E-07 | 290 |
| GO:0000003 | reproduction | Biological process | 3.69E-07 | 27 |
| GO:0022414 | reproductive process | Biological process | 3.69E-07 | 27 |
| GO:0006511 | ubiquitin-dependent protein catabolic process | Biological process | 7.98E-07 | 36 |
| GO:0019941 | modification-dependent protein catabolic process | Biological process | 7.98E-07 | 36 |
| GO:0043632 | modification-dependent macromolecule catabolic process | Biological process | 7.98E-07 | 36 |
| GO:0051704 | multi-organism process | Biological process | 2.85E-06 | 27 |
| GO:0044257 | cellular protein catabolic process | Biological process | 5.78E-06 | 36 |
| GO:0051603 | proteolysis involved in cellular protein catabolic process | Biological process | 5.78E-06 | 36 |
| GO:0030163 | protein catabolic process | Biological process | 9.60E-06 | 37 |
| GO:0043170 | macromolecule metabolic process | Biological process | 3.27E-05 | 416 |
| GO:0044265 | cellular macromolecule catabolic process | Biological process | 7.50E-05 | 37 |
| GO:0007154 | cell communication | Biological process | 1.14E-04 | 55 |
| GO:0033036 | macromolecule localization | Biological process | 1.46E-04 | 44 |
| GO:0009057 | macromolecule catabolic process | Biological process | 2.66E-04 | 38 |
| GO:1901565 | organonitrogen compound catabolic process | Biological process | 2.83E-04 | 38 |
| GO:0006265 | DNA topological change | Biological process | 6.31E-04 | 8 |
| GO:0071702 | organic substance transport | Biological process | 0.003781172 | 45 |
| GO:0006260 | DNA replication | Biological process | 0.00533841 | 15 |
| GO:1901564 | organonitrogen compound metabolic process | Biological process | 0.005571877 | 300 |
| GO:0005634 | nucleus | Cellular component | 2.27E-11 | 66 |
| GO:0099081 | supramolecular polymer | Cellular component | 1.50E-08 | 10 |
| GO:0099080 | supramolecular complex | Cellular component | 1.50E-08 | 10 |
| GO:0099513 | polymeric cytoskeletal fiber | Cellular component | 1.50E-08 | 10 |
| GO:0099512 | supramolecular fiber | Cellular component | 1.50E-08 | 10 |
| GO:0005874 | microtubule | Cellular component | 1.50E-08 | 10 |
| GO:0043226 | organelle | Cellular component | 6.50E-08 | 95 |
| GO:0043229 | intracellular organelle | Cellular component | 6.50E-08 | 95 |
| GO:0043231 | intracellular membrane-bounded organelle | Cellular component | 6.86E-08 | 70 |
| GO:0044430 | cytoskeletal part | Cellular component | 7.64E-08 | 15 |
| GO:0043227 | membrane-bounded organelle | Cellular component | 8.47E-08 | 71 |
| GO:0005856 | cytoskeleton | Cellular component | 1.07E-07 | 15 |
| GO:0015630 | microtubule cytoskeleton | Cellular component | 4.76E-07 | 11 |
| GO:0000786 | nucleosome | Cellular component | 7.29E-05 | 15 |
| GO:0032993 | protein-DNA complex | Cellular component | 7.93E-05 | 15 |
| GO:0044815 | DNA packaging complex | Cellular component | 9.36E-05 | 15 |
| GO:0000785 | chromatin | Cellular component | 1.19E-04 | 15 |
| GO:0044424 | intracellular part | Cellular component | 3.29E-04 | 102 |
| GO:0005622 | intracellular | Cellular component | 3.29E-04 | 102 |
| GO:0044464 | cell part | Cellular component | 0.001128297 | 104 |
| GO:0005623 | cell | Cellular component | 0.001128297 | 104 |
| GO:0044427 | chromosomal part | Cellular component | 0.001407129 | 15 |
| GO:0005694 | chromosome | Cellular component | 0.002360453 | 16 |
| GO:0043228 | non-membrane-bounded organelle | Cellular component | 0.006171625 | 38 |
| GO:0043232 | intracellular non-membrane-bounded organelle | Cellular component | 0.006171625 | 38 |
| GO:0044422 | organelle part | Cellular component | 0.009648032 | 35 |
| GO:0044446 | intracellular organelle part | Cellular component | 0.009648032 | 35 |
| GO:0043531 | ADP binding | Molecular function | 0 | 141 |
| GO:0008234 | cysteine-type peptidase activity | Molecular function | 0 | 89 |
| GO:0008270 | zinc ion binding | Molecular function | 0 | 209 |
| GO:0003676 | nucleic acid binding | Molecular function | 0 | 612 |
| GO:0005515 | protein binding | Molecular function | 0 | 834 |
| GO:0004519 | endonuclease activity | Molecular function | 0 | 115 |
| GO:0004540 | ribonuclease activity | Molecular function | 0 | 115 |
| GO:0004518 | nuclease activity | Molecular function | 2.22E-16 | 118 |
| GO:0004521 | endoribonuclease activity | Molecular function | 3.33E-16 | 115 |
| GO:0016893 | endonuclease activity, active with either ribo- or deoxyribonucleic acids and producing 5'-phosphomonoesters | Molecular function | 3.33E-16 | 104 |
| GO:0005488 | binding | Molecular function | 5.55E-16 | 1916 |
| GO:0016891 | endoribonuclease activity, producing 5'-phosphomonoesters | Molecular function | 5.55E-16 | 104 |
| GO:0004523 | RNA-DNA hybrid ribonuclease activity | Molecular function | 6.66E-16 | 98 |
| GO:0140098 | catalytic activity, acting on RNA | Molecular function | 9.99E-16 | 121 |
| GO:0004857 | enzyme inhibitor activity | Molecular function | 2.09E-13 | 84 |
| GO:0003950 | NAD+ ADP-ribosyltransferase activity | Molecular function | 2.29E-10 | 23 |
| GO:0030234 | enzyme regulator activity | Molecular function | 1.88E-08 | 85 |
| GO:0046914 | transition metal ion binding | Molecular function | 2.44E-08 | 245 |
| GO:0030414 | peptidase inhibitor activity | Molecular function | 6.49E-08 | 35 |
| GO:0061134 | peptidase regulator activity | Molecular function | 6.49E-08 | 35 |
| GO:0061135 | endopeptidase regulator activity | Molecular function | 6.49E-08 | 35 |
| GO:0004866 | endopeptidase inhibitor activity | Molecular function | 6.49E-08 | 35 |
| GO:0098772 | molecular function regulator | Molecular function | 1.32E-07 | 86 |
| GO:0016763 | transferase activity, transferring pentosyl groups | Molecular function | 7.18E-07 | 23 |
| GO:0016788 | hydrolase activity, acting on ester bonds | Molecular function | 7.13E-06 | 134 |
| GO:0070011 | peptidase activity, acting on L-amino acid peptides | Molecular function | 1.16E-05 | 123 |
| GO:0008233 | peptidase activity | Molecular function | 2.88E-05 | 127 |
| GO:0003746 | translation elongation factor activity | Molecular function | 4.22E-05 | 19 |
| GO:0005200 | structural constituent of cytoskeleton | Molecular function | 6.99E-05 | 10 |
| GO:0042393 | histone binding | Molecular function | 4.94E-04 | 8 |
| GO:0008146 | sulfotransferase activity | Molecular function | 0.001547791 | 8 |
| GO:0061505 | DNA topoisomerase II activity | Molecular function | 0.002797553 | 7 |
| GO:0003918 | DNA topoisomerase type II (ATP-hydrolyzing) activity | Molecular function | 0.002797553 | 7 |
| GO:0004779 | sulfate adenylyltransferase activity | Molecular function | 0.004032745 | 5 |
| GO:0004781 | sulfate adenylyltransferase (ATP) activity | Molecular function | 0.004032745 | 5 |
| GO:0008289 | lipid binding | Molecular function | 0.004410531 | 36 |

# Supplementary Table S10 Summary of NBS genes identified in each genome.

| **Species and kinds** | **Abbreviation** | **A17** | **PI464715** | **Msa** | **MsaA** | **MsaB** | **MsaC** | **MsaD** |
| --- | --- | --- | --- | --- | --- | --- | --- | --- |
| **Non-TIR NBS subclass** |  | 469 | 388 | 1,532 | 373 | 390 | 391 | 378 |
| **nRNs** |  | 370 | 326 | 1,343 | 321 | 342 | 352 | 328 |
| BED_CC_NBS_LRR | BCNL | 3 | 8 | 10 | 1 | 1 | 6 | 2 |
| BED-NBS-LRR | BNL | 1 | - | - | - | - | - | - |
| CC-NBS-LRR | CNL | 139 | 117 | 547 | 124 | 148 | 130 | 145 |
| BED-CC-NBS | BCN | - | - | 1 | - | - | 1 | - |
| NBS-LRR | NL | 129 | 113 | 472 | 109 | 122 | 132 | 109 |
| BED-NBS | BN | 1 | - | 1 | - | - | - | 1 |
| NBS only | N | 97 | 88 | 312 | 87 | 71 | 83 | 71 |
| **RN** |  | 99 | 62 | 189 | 52 | 48 | 39 | 50 |
| RPW8-CC-NBS-LRR | RCNL | 74 | 26 | 117 | 32 | 33 | 20 | 32 |
| RPW8-NBS-LRR | RNL | 15 | 11 | 50 | 11 | 10 | 14 | 15 |
| RPW8-CC-NBS | RCN | 8 | 22 | 15 | 8 | 3 | 3 | 1 |
| RPW8-NBS | RN | 2 | 3 | 7 | 1 | 2 | 2 | 2 |
| **TIR NBS subclass** |  | 223 | 101 | 217 | 54 | 54 | 63 | 46 |
| TIR-CC-NBS-LRR | TCNL | 36 | 35 | 62 | 17 | 12 | 19 | 14 |
| TIR_CC_NBS | TCN | 7 | 1 | 3 | - | - | 2 | 1 |
| TIR-NBS-LRR | TNL | 145 | 58 | 131 | 31 | 35 | 39 | 26 |
| TIR-NBS | TN | 35 | 7 | 21 | 6 | 7 | 3 | 5 |
| **Total** |  | 692 | 489 | 1,749 | 427 | 444 | 454 | 424 |

A17: *M. Truncatula*; MsaA: *M. sativa* subgenome 1; MsaB: *M. sativa* subgenome 2; MsaC: *M. sativa* subgenome 3; MsaD: *M. sativa* subgenome 4.
